# Effects of Enterotoxigenic *Escherichia coli* Challenge on Jejunal Morphology and Microbial Community Profiles in Weaned Crossbred Piglets

**DOI:** 10.3390/microorganisms11112646

**Published:** 2023-10-27

**Authors:** Juan Xu, Zhen Jia, Shu Xiao, Cimin Long, Leli Wang

**Affiliations:** 1 Institute of Subtropical Agriculture, Chinese Academy of Sciences, Changsha 410081, China; xujuan1232022@163.com (J.X.); JZYD2733@163.com (Z.J.); xiaoshu_xiao@163.com (S.X.); 2 Hunan Provincial Key Laboratory of Animal Intestinal Function and Regulation, College of Life Sciences, Hunan Normal University, Changsha 410081, China; 3 Tianjin Institute of Industrial Biotechnology, Chinese Academy of Sciences, National Center of Technology Innovation for Synthetic Biology, Tianjin 300308, China

**Keywords:** ETEC, weaning stress, piglets, jejunum

## Abstract

Pathogenic enterotoxigenic *Escherichia coli* (ETEC) is a major cause of bacterial diarrhea in weaning piglets, which are vulnerable to changes in environment and feed. This study aimed to determine the effects of the ETEC challenge on piglet growth performance, diarrhea rate, jejunal microbial profile, jejunal morphology and goblet cell distribution. A total of 13 piglets from one litter were selected on postnatal day 21 and assigned to treatments with or without ETEC challenge at 1 × 10^8^ CFUs, as ETEC group or control group, respectively. On postnatal day 28, samples were collected, followed by the detection of serum biochemical indexes and inflammatory indicators, HE staining, PAS staining and 16S rDNA gene amplicon sequencing. Results showed that the growth performance decreased, while the diarrhea rate increased for the ETEC group. The jejunum is the main segment of the injured intestine during the ETEC challenge. Compared with the control, the ETEC group displayed fewer goblet cells in the jejunum, where goblet cells are more distributed at the crypt and less distributed at the villus. In addition, ETEC piglets possessed higher abundances of the genus *Desulfovibrio*, genus *Oxalobacter* and genus *Peptococus* and lower abundances of the genus *Prevotella* 2, genus *Flavonifractor* and genus *Blautra*. In terms of alpha diversity, Chao 1 and observed features indexes were both increased for the ETEC group. Our study provides insights into jejunal histopathological impairment and microbial variation in response to ETEC infection for weaned piglets and is a valuable reference for researchers engaged in animal health research to select stress models.

## 1. Introduction

Pork production is closely linked to the overall health of the animals, particularly the development and functions of their intestine [1]. Intestinal diseases in weaned pigs are major contributors to antibiotic use in pig farming [2,3,4]. Among these diseases, post-weaning diarrhea stands out as a significant cause of economic loss for the pig industry, resulting from decreased growth rates and the cost of medication [5].

Enterotoxigenic *Escherichia coli* (ETEC) is the primary cause of post-weaning diarrhea. It can be transmitted between piglets through feces, water, and food to exert its pathogenic properties. In actual agricultural production, piglets usually ingest ETEC found in the environment when they arrive in the nursery, especially derived from mammary glands and from the farrowing room or the pen environment [6]. This common pathogen affects not only piglets in animal husbandry but also children in developing countries and regions [7,8]. The heat-labile toxin and heat-stable toxin from ETEC were reported to stimulate the intestinal lining and cause watery diarrhea during infection [9]. Pathogenic *E. coli* poses a significant threat to human health through food- and water-borne transmission from domestic pigs to humans [10]. A previous study conducted in Europe examined the prevalence of fimbrial and toxin genes in *E. coli* among 873 samples associated with post-weaning diarrhea. Researchers discovered that *E. coli* isolates containing both fimbrial and toxin genes accounted for 52.5% of the 339 isolates analyzed. Of these, 94.9% were classified as ETEC, with the most common virotype being F4 [11]. This highlights the need for continued research and intervention strategies to mitigate the effects of ETEC on both animal and human health.

The intestinal morphology is an important indicator of intestinal health [12]. Villous height is related to the growth performance and the digestion and absorption capacity in piglets [13]. In addition, crypt length could affect the migration of cells in infectious diarrhea [14]. There is evidence that weaning stress could cause significant changes in intestinal morphology [15].

The mechanisms involved in the host response to ETEC adherence and colonization in piglets are not well understood. Therefore, the study aimed to investigate the effects of the ETEC challenge on different segments of the intestine in weaning pigs. Additionally, the study intended to examine the microbial community profiles and goblet cell count in the jejunum of piglets from a litter, with and without ETEC challenge.

To achieve this, we established an ETEC-induced diarrhea model in experimental piglets through gavage. The intestinal morphology and contents, as well as serum-related factors, using internal and external indicators, were evaluated. Furthermore, we sequenced and compared the microbial communities in the piglet intestines at the cellular and molecular levels to identify the primary metabolites involved in intestinal microbes. Overall, this study aimed to gain a better understanding of the host response to ETEC in piglets and provide insights into the morphological and phenotypic impacts of the ETEC challenge.

## 2. Materials and Methods

The piglet trial protocol and procedures performed in this study were approved by the Animal Care and Use Committee at Guangdong Academy of the Institute of Subtropical Agriculture, Chinese Academy of Sciences.

### 2.1. Preparation of Enterotoxigenic Escherichia coli F4

The *Escherichia coli* F4-producing strain W25K (O149:K91, K88ac; LT, ST, EAST) [16] was gifted from Prof. Wenkai Ren, which was isolated from a diarrheal pig and preserved in 25% (*v*/*v*) glycerol broth at −80 °C at the College of Life Science, Hunan Normal University, China. The ETEC strain was streaked on Luria-Bertani (LB) agar medium from frozen stock and cultured anaerobically at 37 °C overnight. Subsequently, 2 mL sterile LB liquid broth was inoculated with a single ETEC colony from the agar plate and in an orbital thermomixer at 800 rpm and 37 °C overnight. Next, one milliliter of the fresh overnight was used to inoculate 500 mL LB liquid broth. Following serial dilution, the concentration of fermentation broth was determined by counting colony-forming units. Sterile LB liquid was used as the diluent to achieve the targeted ETEC concentration, which was 1 × 10^8^ colony-forming units (CFUs)/mL based on a previous study [17]. Immediately, the culture was transported to the experimental farm with ice packs.

### 2.2. Animals and Experimental Design

**Figure 1:**
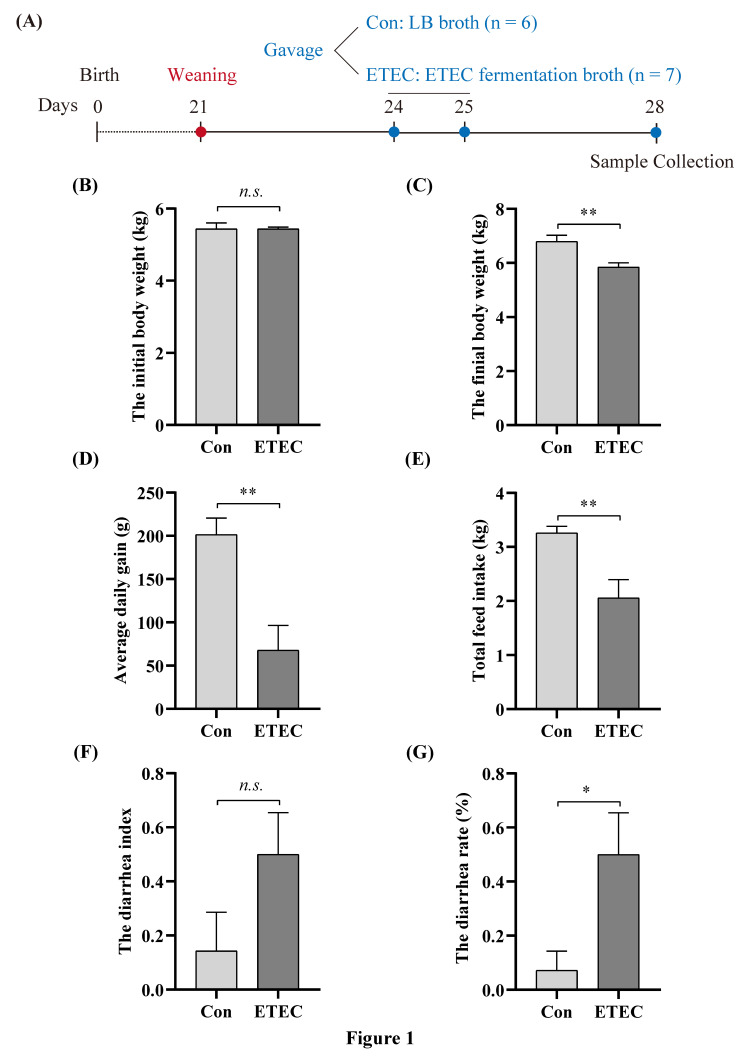
Effects of ETEC challenge on growth performance and fecal score. (**A**) Experimental outline in weaned piglets. (**B**) The initial body weight at 21 days. (**C**) The final body weight at 27 days. (**D**) Average daily gain from 21 to 27 days. (**E**) Total feed intake from 21 to 27 days. (**F**,**G**) The diarrhea index and diarrhea rate between control group and ETEC group after ETEC gavage. Values of the bars stand for significant differences using two-tailed unpaired *t*-tests at *p* < 0.05. *, *p* < 0.05; **, *p* < 0.01; *n.s.*, no significant differences.

Thirteen crossbred piglets (*Duroc* × *Landrace* × *Yorkshire*, DLY) with an average body weight of 5.49 ± 0.33 kg were weaned at 21 days and selected from a litter, which was obtained from the Xinwufeng company at Changsha and housed individually in a temperature-controlled room (Figure 1A). Piglets were randomly distributed into two groups. (1) Sham-challenged control: six piglets were administrated with 20 mL of sterile LB liquid medium at 24 days and 25 days. (2) ETEC group: seven piglets were orally administrated with 20 mL ETEC W25K (1 × 10^8^ CFU/mL) for two consecutive days. A corn–soybean meal basal diet in a mash form was formulated to meet or exceed the Nation Research Council recommendations (NRC, 2012) for weaning piglets. Piglets were housed in separate pens and free to obtain water and feed throughout the experiment.

Fecal consistency (0 = normal feces; 1 = soft feces; 2 = fluid feces with yellowish color; 3 = watery and projectile feces) was scored at 24, 25, 26 and 27 days as described previously [18]. And the diarrhea index and diarrhea incidence were calculated in accordance with a previous report [19]: diarrhea index = sum of diarrhea scores for each group of piglets during the trial period/(number of days tested × number of piglets per group); diarrhea incidence (%) = number of piglets with diarrhea per treatment during the trial period/(number of days tested × number of piglets per group) × 100%.

### 2.3. Sample Collection

Piglets were individually weighed beginning on day 21 of the experiments. After being fasted overnight, piglets were euthanized by intravenous administration of 150 mg/kg body weight pentobarbital (Kela, Hoogstraten, Belgium), and then sacrificed at 28 days. The blood samples with approximately ten milliliters were collected from each piglet through venipuncture of the anterior vena cava. About 2 cm of intestinal tissue segments were instantly isolated from the same parts of the ileum, duodenum, jejunum and colon cautiously to avoid squeezing and fixed in 4% paraformaldehyde for intestinal morphology analysis. The leftover intestinal segments and digesta samples were transported to liquid nitrogen rapidly.

### 2.4. Serum Parameters Analysis

The collected blood samples were separated after centrifugation at 3000× *g* for 10 min to obtain serum. The serums were kept at −80 °C before analysis. Serums were measured using commercial reagent kits according to the manufacturer’s instructions (Jiancheng Bioengineering Institute, Nanjing, China) and identified by the TBA-120FR Automatic Biochemistry Radiometer (Hitachi Co., Tokyo, Japan). These parameters were also analyzed, including α-amylase (α-AMY); albumin (ALB); alkaline phosphatase (ALP); alanine aminotransferase (ALT); aspartate aminotransferase (AST); blood urea nitrogen (BUN); cholestenone (CHO); creatinine (CREA); direct bilirubin (DBIL); globulin (GLO); glucose (GLU); high-density lipoprotein cholesterol (HDL-C); indirect bilirubin (IBIL); lactate dehydrogenase (LDH); low-density lipoprotein cholesterol (LDL-C); total bilirubin (TBIL); triglyceride (TG); and total protein (TP). Concentrations of immunoglobulins M (IgM), immunoglobulins G (IgG), interleukin1-β (IL-1β) and interleukin-6 (IL-6) in the serum were determined through commercial quantitative enzyme-linked immunosorbent assay kits according to the manufacturer’s instructions.

### 2.5. Hematoxylin and Eosin Staining

The histomorphology of intestinal segments was determined according to previous studies [20,21]. All samples were fixed in 4% paraformaldehyde solution for 48 h and then embedded in paraffin. Sections of 5 μm were cut on a microtome (RM2235; Leica; Los Angeles, CA, USA) and then stained with hematoxylin–eosin to analyze at least 22 intact well-oriented crypt-villus units in each section in every piglet. Imagines were acquired using a light microscope (Leica DM3000; Leica Microsystem; Wetzlar, Germany) with 10× magnification. Villus height and crypt depth were blindly determined through Image Pro-Plus Software (version 6.0; Media Cybernetics, Silver Spring, MD, USA).

### 2.6. Periodic Acid–Schiff Staining

The goblet cells in the jejunum were counted according to a previous study [20]. Using a PAS staining reagent kit (C0142M, Beyotime, Shanghai, China), periodic acid–Schiff (PAS) staining was performed according to the protocol. With the PAS and Alcian Blue, the positively stained goblet cells were visualized and separately counted based on the location of villi and crypts. Images were acquired with a computer-assisted microscope (Leica DM3000; Leica Microsystem) with 10× magnification. Goblet cells with PAS staining were counted by a blinded observer.

### 2.7. 16S rDNA Gene Amplicon Sequencing

The digesta sample of jejunum was brought to the laboratory under the freezing of liquid nitrogen and was stored at −80 °C before analysis. Using the E.Z.N.A.^®^ Stool DNA Kit (Omega Bio-tek, Inc., Norriston, GA, USA), total bacterial DNA was extracted as instructed [22]. Then, the DNA integrity was assessed by electrophoresis on 1.5% agarose gel. The 16S rDNA hypervariable region V3-V4 was amplified using a pair of previously used primers 338F (5′-ACTCCTACGGGAGGCAGCAG-3′) and 806R (5′-GGACTACHVGGGTWTCTAAT-3′) [23]. The volume of polymerase chain reaction mixtures contains *TransStart* FastPfu buffer and DNA polymerase.

After purification from agarose gel, amplicons were pooled and performed double-ended sequence on the Illumina MiSeq PE300 platform (Illumina, San Diego, CA, USA). Reads were distinguished by the primers and barcodes, before adjustment of the sequence direction [24]. For sequences denoising, an open-source software package DADA2 (https://github.com/benjjneb/dada2 (accessed on 20 November 2021)) was used [25]. Using the UPARSE software, operational taxonomic units (OTUs) were calculated at 97% sequence similarity [26]. The taxonomy of representative sequence was aligned against the SILVA version 132 database [27]. Using QIIME2 software (version: 2021.8), the alpha diversity indexes and the bata diversity indexes were conducted [28]. The beta diversity was processed by principal component analysis based on Jaccard distance.

### 2.8. Statistical Analysis

Data were presented as means ± standard error of the mean (SEM). Differences between the ETEC and control groups were considered significant at *p* < 0.05. Data analysis was conducted using SPSS software (version 19.0; IBM Corp., Chicago, IL, USA). Data and graph presentation were performed using GraphPad Prism software (version 8.3; San Diego, CA, USA). Significant differences were tested using a two-tailed unpaired *t*-test. Taxonomic differences were tested using the Wilcoxon signed-rank test on OTUs aggregated data. *, *p* < 0.05; **, *p* < 0.01, ***, *p* < 0.001 indicate significant differences between samples; *n.s.*, indicates not significant.

## 3. Results

### 3.1. Effects of ETEC Challenge on Growth Performance and Fecal Score

ETEC W25K is an O149:K88 serotype strain, which was isolated from a diarrheal piglet [16,29]. Thirteen weaned piglets were randomly separated into a control group and an ETEC group (Figure 1A). In comparison to the control group, the final body weight in the ETEC-treated group decreased by 13.83% (*p* < 0.05). The challenge of ETEC caused serious body weight loss in weaned piglets (Figure 1B,C). In addition, the ETEC challenge reduced the average daily gain (Figure 1D) and total feed intake (Figure 1E) in the piglets compared to the control group. There was no significant difference in the diarrhea index between the two groups (Figure 1F). However, the diarrhea rate was markedly increased (*p* < 0.05) after the ETEC challenge (Figure 1G).

### 3.2. Effect of ETEC Challenge on Serum Biochemistry

**Figure 2:**
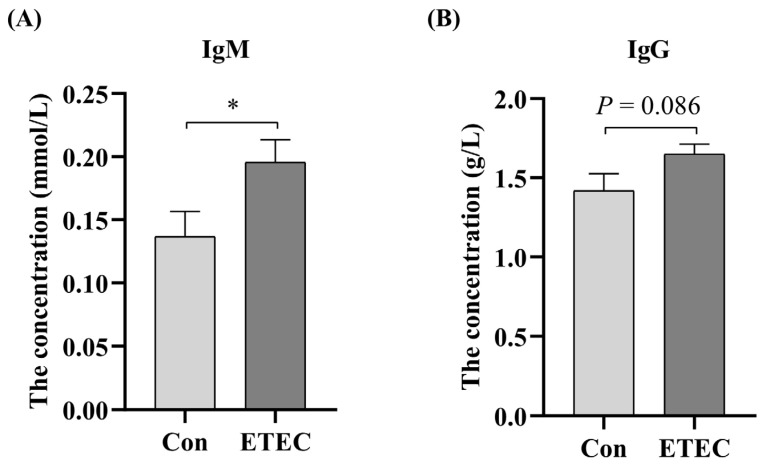
Effect of ETEC challenge on the levels of (**A**) IgM and (**B**) IgG in serum. Values of the bars stand for significant differences using two-tailed unpaired *t*-tests at *p* < 0.05. *, *p* < 0.05.

Serum biochemical parameters were detected to further explore the effect of the ETEC challenge on weanling piglets. ETEC challenge elevated the concentrations of IgM in serum (*p* < 0.05) (Figure 2A). It is also worth noting that compared with the control group, serum IgG (*p* = 0.086) in the ETEC group tended to increase (Figure 2B). However, there was no significant difference in other serum biochemical parameters between the control group and the ETEC group (Table S1).

### 3.3. Effect of ETEC Challenge on Intestinal Segments

**Figure 3:**
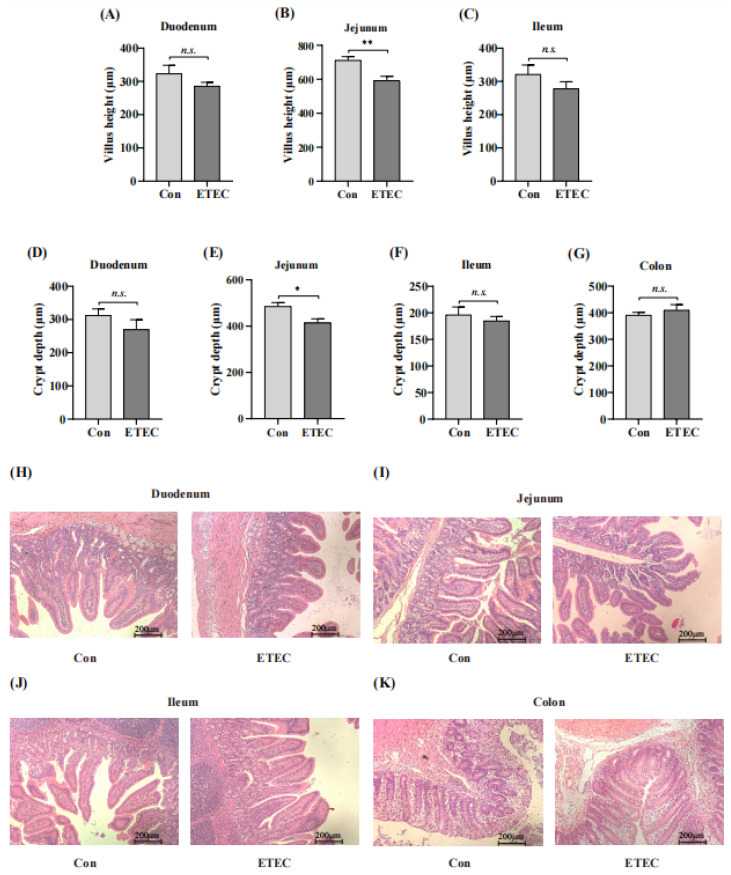
Effect of ETEC challenge on the intestinal morphology of (**A**–**C**) villus height in small intestinal, (**D**–**F**) crypt depth in small intestinal and (**G**) crypt depth in colon. The representative microphotographs of hematoxylin–eosin stained intestinal sections in (**H**) duodenum, (**I**) jejunum, (**J**) ileum and (**K**) colon were also shown. Values of the bars stand for significant differences using two-tailed unpaired *t*-tests at *p* < 0.05. *, *p* < 0.05; **, *p* < 0.01; *n.s.*, no significant differences. Scale bars, 200 μm.

To further investigate the effects of ETEC challenge on the intestine in weanling piglets, the morphologies of different intestinal segments were measured by hematoxylin–eosin staining including duodenum, jejunum, ileum and colon (Figure 3A–K). Compared with the control group, shorter villus height was observed in the jejunum (*p* < 0.01) for the ETEC challenge (Figure 3A–C,I). In addition, jejunal crypt depth was also significantly decreased in the ETEC group (*p* < 0.05) (Figure 3E,I), whereas there was no significant difference in crypt depth among these intestine segments. Collectively, the jejunum is the main segment of the injured intestine in the ETEC challenge with the low villus height [30].

### 3.4. Effect of ETEC Challenge on Goblet Cell in Jejunum

**Figure 4:**
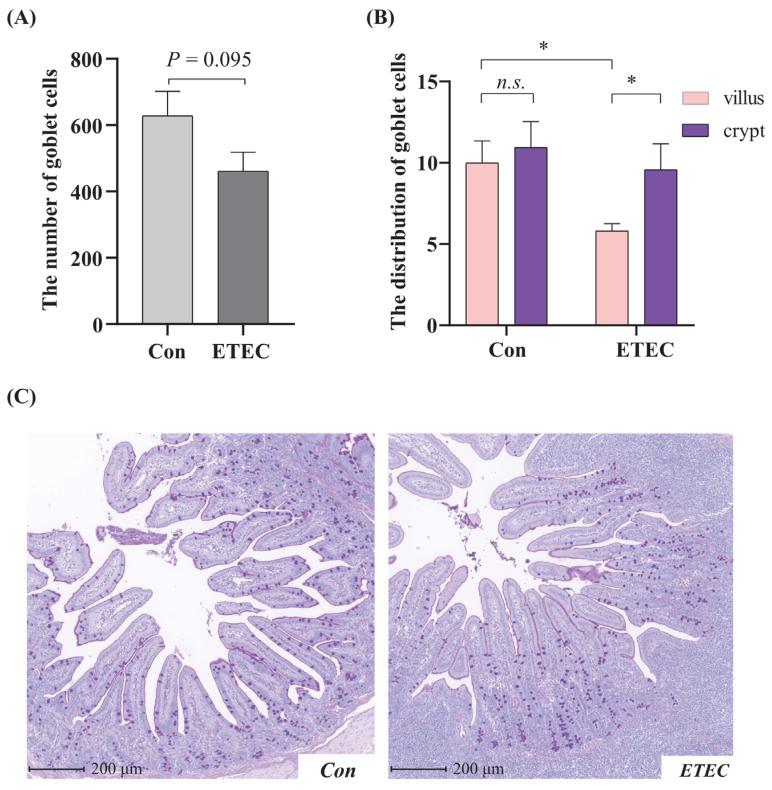
Effect of ETEC challenge on goblet cell. (**A**) The total number of goblet cells in jejunum. (**B**) The distribution of goblet cells from villus to crypt in jejunum. Values of the bars stand for significant differences using two-tailed unpaired *t*-tests at *p* < 0.05. *, *p* < 0.05; *n.s.*, no significant differences. (**C**) Representative microphotographs of PAS-stained jejunal sections from the control group and ETEC group. Scale bars, 200 μm.

The number of goblet cells in the jejunum was slightly decreased (*p* = 0.095) by ETEC challenge (Figure 4A). To quantify this apparent change in cellular distribution, goblet cells in the villus and crypts were enumerated. Following the ETEC challenge, there was a more uneven distribution of generally uniformly sized goblet cells along the jejunal villi of the ETEC group, while the distributions in the control were even (Figure 4B,C). Specifically, the number of jejunal villus goblet cells in the ETEC group was less than that in the control group (*p* < 0.05). In the ETEC group, the number of goblet cells within crypts was obviously higher than that within villi (*p* < 0.05). Together, the ETEC challenge changed the count and distribution of goblet cells in the jejunum.

### 3.5. Effect of ETEC Challenge on Jejunal Microbiome

**Figure 5:**
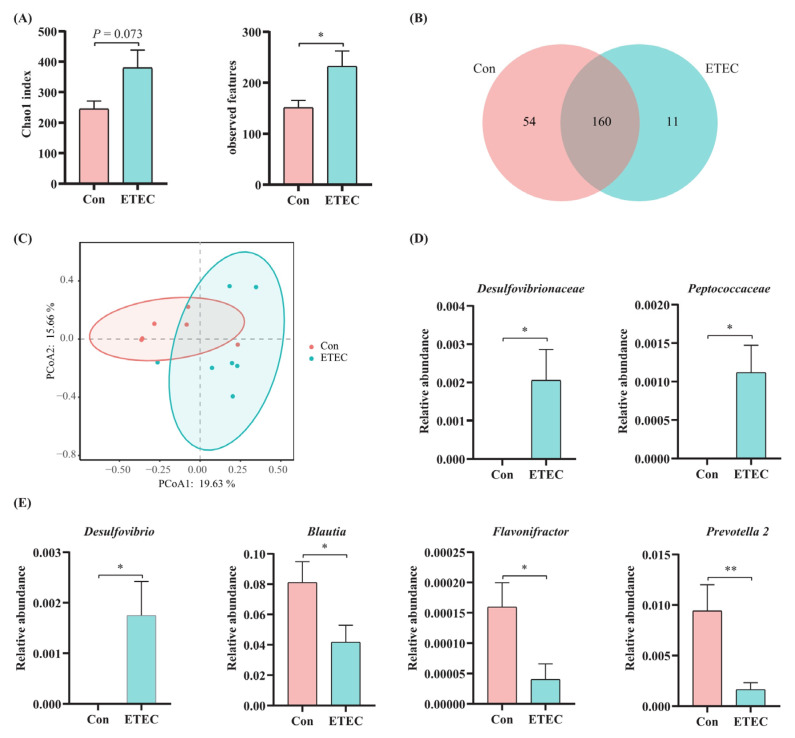
The 16S rDNA gene sequencing analysis of ETEC challenge on jejunal microbiome. (**A**) Alpha diversity comparisons of the gut microbiome, including Chao 1 index and observed features. (**B**) Venn diagram showing the coincidence of operational taxonomic units between the two groups. The total number of goblet cells in jejunum. (**C**) Scatterplots of NMDS analysis depicting differences in the bacterial community structure on genus level between the control group and ETEC group. (**D**) The relative abundances of families with significant differences. (**E**) The relative abundances of genera with or without ETEC challenge. Values of the bars stand for significant differences using two-tailed unpaired *t*-tests at *p* < 0.05. *, *p* < 0.05; **, *p* < 0.01.

To explore the influence of the ETEC challenge on the jejunal microbiota, we sequenced the V4 hypervariable region of the 16S rDNA gene to identify the jejunal bacterial community and composition. In terms of alpha diversity, Chao 1 and observed features indexes were both increased for the ETEC group than for the control group (i.e., control vs. ETEC: 245.31 ± 57.76 vs. 379.73 ± 142.83 and 151.00 ± 32.75 vs. 232.00 ± 74.38 for Chao 1 and observed features), indicating there was more abundant bacteria in ETEC group (Figure 5A). The shared and specific genera were shown in a Venn diagram (Figure 5B). As for beta diversity, the PCoA plot showing the dissimilarity of the microbial community also revealed different structures between ETEC and control piglets, and the ANOSIM for differences between the two groups was significant (*R* = 0.331, *p* = 0.019, *Q* = 0.0285). The observation was supported by Jaccard distances (Figure 5C). At the family level, compared with the control group, ETEC-challenged piglets showed a significant increase in the relative abundances of *Desulfovibrionaceae* (lineage: *Thermodesulfobacteriota*; *Desulfovibrionia*; *Desulfovibrionales*) (*p* = 0.039) and *Peptococcaceae* (lineage: *Terrabacteria* group; *Bacillota*; *Clostridia*; *Eubacteriales*) (*p* = 0.014) (Figure 5D). At the genus level, the relative abundance of the genus *Desulfovibrio* (lineage: *Thermodesulfobacteriota*; *Desulfovibrionia*; *Desulfovibrionales; Desulfovibrionaceae*) (*p* = 0.036) was upregulated by ETEC challenge. Besides, the piglets in ETEC-treated group showed a decreased abundance of genus *Blautia* (lineage: *Terrabacteria* group; *Bacillota*; *Clostridia*; *Eubacteriales*; *Lachnospiraceae*) (*p* = 0.046), genus *Flavonifractor* (lineage: *Terrabacteria* group; *Bacillota*; *Clostridia*; *Eubacteriales*; *Oscillospiraceae*) (*p* = 0.026) and genus *Prevotella* 2 (lineage: FCB group; *Bacteroidota/Chlorobiota* group; *Bacteroidota*; *Bacteroidia*; *Bacteroidales*; *Prevotellaceae*) (*p* = 0.0095), compared to the control group (Figure 5E).

## 4. Discussion

One of the biggest challenges faced by the global pig breeding industry is diarrhea [31,32], which is characterized by frequent watery feces, leading to retarded growth, damaged gut health, increased morbidity, and mortality [33]. During the stressful weaning period, piglets are particularly susceptible to infection by various pathogens, due to their fragile gastrointestinal tracts and weakened immunity coupled with abrupt changes in dietary structure from easily digestible milk to less digestible solid feed [34,35,36]. Among the pathogens responsible for diarrhea in nursery and weaning pigs, some *E. coli* species are common causal agents [37,38]. ETEC is the most prevalent pathogenic bacterium among the six recognized diarrheagenic *Escherichia coli* classifications (enteropathogenic, enterotoxigenic, Shiga toxin-producing, enteroinvasive, enteroaggregative, and diffuse-adhering *E. coli*) [39,40].

**Figure 6:**
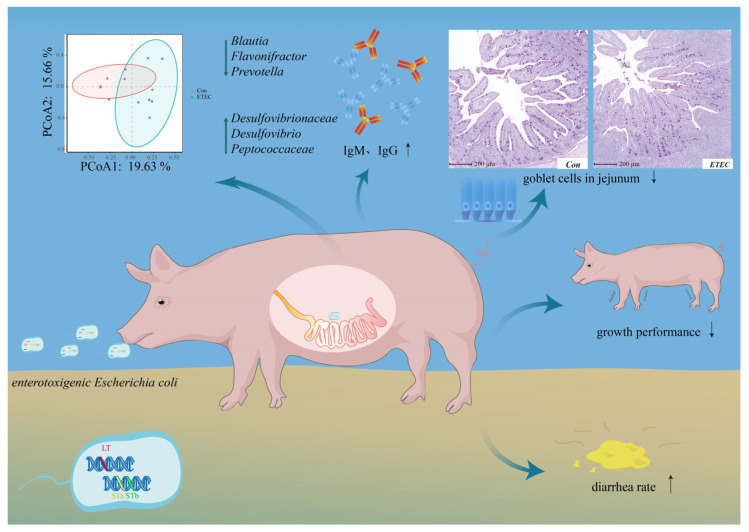
A schematic picture of ETEC infection in weaned piglets, which display poor growth performance, serious diarrhea symptoms and inflammatory response, impaired gut morphology, decreased goblet cells and microbial variation in this study.

ETEC infection caused a high diarrhea rate in this study (Figure 6). The virulent strain ETEC W25K, belongs to the O149 serotype, carrying the F4 (K88) adhesion. ETEC strains are known to excrete heat-labile enterotoxin and heat-stable enterotoxin, which induce water and electrolyte loss from the intestine [41,42,43]. After ETEC colonizes the small intestine, these enterotoxins are likely to penetrate the blood through the damaged intestinal epithelium [44,45,46].

In addition, colonization and toxins of ETEC induced an immune response in the weaned piglets. Serum biochemical parameters and immunoglobulin concertation can reflect the physiological and immune conditions of piglets [47,48]. An increase in serum IgM was observed in our study, which was considered to be related to the rapid and intense immune response and the alteration in the gut microbiota [49,50]. IgM is the first antibody produced during the initial response and can stimulate inactivated B cells, leading to Ig class switching recombination to produce different isotype antibodies [51]. A high level of IgM in serum samples is an important criterion for early diagnosis of infectious disease infection [52,53]. In line with our study, Liu et al. reported [50] that mice administered with ETEC RNA by intramuscular injection and oral route significantly promoted the production of serum IgM. However, in some studies [54,55], *E. coli* could induce a serum IgG response but not IgM induction in mouse models. Hurtado et al. reported that the human antimicrobial peptide LL-37 promoted B cell activation and increased IgM and IgG production [56]. Similarly, Shan et al. reported that artificially synthesized antimicrobial peptides increased serum IgG and IgM in weanling piglets [57]. Additionally, Wang et al. even showed that the levels of serum IgG and IgM increased with the reduced abundance of *Enterobacteriaceae* in pigs treated with fermented feed [58], which also revealed the correlation between the gut microbiota and serum indicators. IgM is a vital anti-inflammatory component and elevated IgM content indicates better immunity [59]. The increased concentration of IgG is also reflected in a better immunological response and health for the lactating sows [60]. The increase in serum IgG and IgM concentrations elicited by ETEC invasion in our study might help eliminate invaders and neutralize toxins through specific binding with corresponding antigens in humoral immunity [61].

Many studies have shown that ETEC impairs the health status of jejunum [9]. During infection, ETEC could colonize at small intestine of hosts and secrete exotoxins [62]. For instance, Wu et al. reported that diarrheal piglets displayed elevated levels of 4-aminobutyic acid and glycine in the jejunum [16]. Similarly, Ren et al. [9] analyzed the jejunum protein profile and found that NF-κB and MAPK pathways were inhibited in piglets infected with ETEC, and calcineurin B homologous protein 1 was downregulated. This protein plays a critical role in sodion transport in the intestinal epithelium [63,64,65]. Intestinal morphology is commonly used to measure the health of intestinal epithelium and the ability to digest and absorb nutrients, which is also an important index in determining the diarrhea sensitivity of piglets [66,67,68,69]. As expected, our study found that the ETEC challenge led to shorter villus height in the jejunum. A previous study reported that lipopolysaccharide challenge in weanling piglets led to various morphological changes in the jejunum, such as necrosis and decreased villus height [70]. As the intestinal villus height becomes shorter, the absorption area of the intestine decreases, which affects nutrient absorption, which could explain the worse growth performance in our study [71].

Our study further investigated the effects of ETEC invasion on goblet cells in the jejunum. The villous epithelium mainly consists of absorptive enterocytes and specialized secretory cells, such as goblet cells [72]. Intestinal goblet cells play an important role in the elimination of undigested food, microbes, and microbial products [73], by secreting mucins to form mucus layers critical for maintaining the integrity of the intestinal epithelium [72]. In our study, the number of goblet cells in the jejunum was slightly decreased by ETEC challenge and more goblet cells were distributed in the jejunal crypts. Loss of intestinal goblet cells is implicated in enteric disease [72]. Clinically, a decrease in goblet cell numbers in premature infants led to the propensity for adherence of pathogenic organisms and their subsequent translocation [74]. It is also reported that ETEC infection in mice activated the mRNA expression of Mucin 2, which is expressed by goblet cells [75]. In broilers, delayed access to feed can inhibit small intestine development and change the distribution of goblet cells in the jejunal villi [76]. However, Almeida et al. reported that the challenge with a pathogenic *E. coli* increased the number of goblet cells [77], which is inconsistent with our results.

Apart from impaired goblet cell function, structural disruption of the gut microbiota is an etiological factor in enteric diseases [78]. The 16S rDNA approach has been widely applied to investigate microbial alternations in pathogen-infective diseases [79,80]. After early weaning, the gut microbiome is not yet well-established and not yet mature under the invasion of pathogenic bacteria [81]. In terms of alpha diversity, both Chao 1 and observed feature indexes were increased in the ETEC group. Consistent with our study, Yue et al. found that the scores of observed features and the Chao 1 index of gut microbiota in antipsychotic-induced constipation patients were higher than those in non-constipation patients [82]. However, the fecal microbial richness of cows with mastitis significantly decreased after cephalosporin treatment, which was indicated by the decreased Chao 1 index [83]. At the family level, the relative abundances of *Desulfovibrionaceae* and *Peptococcaceae* in the jejunum of weaned piglets in the ETEC challenge group were significantly enriched compared with the control in our study. *Desulfovibrio* and *Peptococcaceae* are known as opportunistic pathogens that are positively related to gut inflammation [84,85]. A recent study showed that after treatment with melatonin, an anti-inflammatory agent and antioxidant, the Chao index and the proportions of harmful bacterial genera *Desulfovibrio* and *Peptococcaceae* were decreased in oxazolone-induced colitis mice [85]. Moreover, at the genus level, the relative abundance of *Desulfovibrio* in the jejunum of weaned piglets in the ETEC challenge group was significantly enriched. *Desulfovibrio* is known as an opportunistic pathogen [86] and is associated with ulcerative enteritis and immune system imbalance [87]. At the genus level, the relative abundances of *Prevotella 2*, *Flavonifractor* and *Blautia* in the jejunum of weaned piglets in the ETEC challenge group were significantly decreased. Sankaranarayanan et al. demonstrated that *Flavonifractor plautii* was able to degrade quercetin into metabolites with anticancer properties and has the potential to be developed into probiotics to improve gut health [88]. In addition, maternal dietary resveratrol was demonstrated to increase the abundance of *Flavonifractor* to alleviate weaning-associated intestinal inflammation and diarrhea in offspring [89]. In accordance with our study, the decreased relative abundance of *Blautia* was also observed in the fecal microbiota of diarrheic neonatal piglets [90]. Bin et al. reported that piglets with ETEC-induced diarrhea had a lower percentage of *Prevotella* in the feces [91].

## 5. Conclusions

In summary, ETEC infection decreased growth performance, induced diarrhea and inflammatory response, damaged gut morphology, and decreased the goblet cells. Our study provides insights into jejunal histopathological impairment and microbial variation in response to ETEC infection for weaned piglets. These results could be useful in selecting targets for intervention during ETEC invasion.

## Data Availability

The datasets analyzed during this study are available from the corresponding author upon reasonable request. The raw reads were deposited into the NCBI database with accession number PRJNA926361.

## Supplementary Materials

The following supporting information can be downloaded at: https://www.mdpi.com/article/10.3390/microorganisms11112646/s1, Table S1: the effect of ETEC treatment on serum biochemistry of weanling piglets.
