# Supplementary material for: Effects of Enterotoxigenic Escherichia coli Challenge on Jejunal Morphology and Microbial Community Profiles in Weaned Crossbred Piglets
Source: Microorganisms. 2023 Oct 27;11(11):2646. doi: 10.3390/microorganisms11112646 (PMC10672776; doi:10.3390/microorganisms11112646)
Supplement: Supplementary file 1 [file microorganisms-11-02646-s001.zip › microorganisms-2607553-supplementary.pdf]

Table S1. The effect of ETEC treatment on serum biochemistry of weanling piglets.

Items	Control	ETEC	*P* value
α-AMY, U/L	2092.45 ± 90.42	2284.27 ± 167.51	0.358
ALB, g/L	32.32 ± 0.70	33.30 ± 1.11	0.490
ALP, U/L	385.50 ± 41.22	421.86 ± 38.10	0.530
ALT, U/L	36.33 ± 3.81	34.57 ± 4.43	0.773
AST, U/L	58.33 ± 14.42	61.00 ± 16.46	0.764
BUN, μmol/L	1.24 ± 0.15	1.46 ± 0.10	0.265
CHO, mmol/L	2.21 ± 0.10	2.47 ± 0.13	0.150
CREA, μmmol/L	83.50 ± 2.40	90.14 ± 3.85	0.188
DBIL, μmmol/L	0.65 ± 0.442	0.86 ± 0.65	0.525
GLO, g/L	12.40 ± 0.85	11.17 ± 1.888	0.585
GLU, mmol/L	5.84 ± 0.38	5.45 ± 0.69	0.640
HDL-C, mmol/L	0.86 ± 0.06	0.94 ± 0.06	0.428
IBIL, μmmol/L	0.38 ± 0.31	0.60 ± 0.46	0.344
IL-1β, pg/ml	6.71 ± 0.37	6.71 ± 0.29	0.996
IL-6, pg/ml	2.92 ± 0.17	3.30 ± 0.28	0.291
LDH, U/L	841.67 ± 30.11	840.06 ± 64.10	0.983
LDL-C, mmol/L	1.07 ± 0.04	1.23 ± 0.07	0.103
TBIL, μmmol/L	1.03 ± 0.70	1.46 ± 1.10	0.433
TG, mmol/L	0.27 ± 0.03	0.29 ± 0.05	0.825
TP, g/L	44.72 ± 0.90	45.76 ± 1.79	0.633

Note. The two tailed unpaired t-test was used to test for significant differences at 0.05. α-AMY = α-amylase; ALB = albumin; ALP = alkaline phosphatase; ALT = Alanine aminotransferase; AST = Aspartate aminotransferase; BUN = blood urea nitrogen; CHO = cholestenone; CREA = creatinine; DBIL = direct bilirubin; GLO = globulin; GLU = glucose; HDL-C = high density lipoprotein cholesterol; IBIL = indirect bilirubin; IL-1β = Interleukin-1β; IL-6 = Interleukin-6; LDH = lactate dehydrogenase; LDL-C = low density lipoprotein Cholesterol; TBIL = total bilirubin; TG = triglyceride; TP = total protein.
